# A Novel Microfluidic Method Utilizing a Hydrofoil Structure to Improve Circulating Tumor Cell Enrichment: Design and Analytical Validation

**DOI:** 10.3390/mi11110981

**Published:** 2020-10-30

**Authors:** Gürhan Özkayar, Ege Mutlu, Şebnem Şahin, Yağmur Demircan Yalçın, Taylan Töral, Haluk Külah, Ender Yildirim, Özge Zorlu, Ebru Özgür

**Affiliations:** 1 Mikro Biyosistemler A.Ş., ODTÜ Teknokent MET Yerleskesi, No:280/B/10, Ankara 06530, Turkey; gozkayar@gmail.com (G.Ö.); ege.mutlu@mikrobiyo.com.tr (E.M.); sebnem.sahin@mikrobiyo.com.tr (Ş.Ş.); yagmur.demircan@mikrobiyo.com.tr (Y.D.Y.); taylan.toral@mikrobiyo.com.tr (T.T.); kulah@metu.edu.tr (H.K.); yender@metu.edu.tr (E.Y.); ozge.zorlu@mikrobiyo.com.tr (Ö.Z.); 2 Department of Electrical and Electronics Engineering, Middle East Technical University (METU), Ankara 06530, Turkey; 3 Department of Mechanical Engineering, Middle East Technical University (METU), Ankara 06530, Turkey

**Keywords:** circulating tumor cells, cancer, liquid biopsy, microfluidics, inertial particle focusing

## Abstract

Being one of the major pillars of liquid biopsy, isolation and characterization of circulating tumor cells (CTCs) during cancer management provides critical information on the evolution of cancer and has great potential to increase the success of therapies. In this article, we define a novel strategy to effectively enrich CTCs from whole blood based on size, utilizing a spiral microfluidic channel embedded with a hydrofoil structure at the downstream of the spiral channel. The hydrofoil increases the distance between the streams of CTCs and peripheral blood cells, which are already distributed about two focal axes by the spiral channel, thereby improving the resolution of the separation. Analytical validation of the system has been carried out using Michigan Cancer Foundation-7 (MCF7) breast cancer cell lines spiked into blood samples from healthy donors, and the performance of the system in terms of white blood cell (WBC) depletion, CTC recovery rate and cell viability has been shown in single or two-step process: by passing the sample once or twice through the microfluidic chip. Single step process yielded high recovery (77.1%), viable (84.7%) CTCs. When the collected cell suspension is re-processed by the same chip, recovery decreases to 65.5%, while the WBC depletion increases to 88.3%, improving the purity. Cell viability of >80% was preserved after two-step process. The novel microfluidic chip is a good candidate for CTC isolation applications requiring high recovery rate and viability, including functional downstream analyses for variety of cancer types.

## 1. Introduction

Despite emerging detection technologies and new therapy regimes, cancer is the main health issue all over the world. It is the second leading cause of death worldwide and the incidence rate is on the rise [1]. Success in surgery and treatment depends highly on early detection. On the other hand, precise monitoring of the therapeutic response, detection of resistance mutations and application of more precise, personalized therapies are crucial to increase the treatment efficiency in advanced stages. 

The current standard of care for cancer diagnosis is fine-needle or surgical solid biopsy, where a section of tumor tissue is analyzed for the biochemical and molecular characterization to stratify the cancer and identify cancer-linked mutations. However, there are many constraints associated with standard solid biopsies. These include insufficiency of the tumor sample for extensive molecular characterization, inaccessibility of tumor site especially in metastatic cases, and limited information on intra- and inter-tumor heterogeneity, not to mention its complicated and highly invasive nature, causing patient discomfort and limit frequent sampling to monitor tumor evolution.

The approach brought about by “liquid biopsy”, detection of circulating tumor biomarkers from body fluids for cancer diagnostics, aims to overcome difficulties and shortcomings associated with traditional solid biopsy and has a great potential to transform the lives of cancer patients [2]. The technology offers obvious advantages, like:

(i) non-invasive tumor sampling which significantly reduces the patients’ discomfort,
(ii) frequent sampling to monitor therapy efficacy and evolution of resistance mutations, paving the way for a more precise disease management (through the selection of more appropriate targeted therapies, which avoids the side-effects and costs associated with inappropriate chemotherapy),
(iii) better representation of the spatial and temporal heterogeneity of the solid tumors.

The liquid biopsy field covers the detection of a variety of circulating tumor biomarkers from bodily fluids. These include circulating tumors cells (CTCs), tumor-derived nucleic acids (ctDNA), exosomes, and tumor-educated platelets. All of these biomarkers give invaluable and complementary information on tumor progression [3,4]. 

CTCs are cancer cells that detach from the primary or metastatic tumors and shed into blood through intravasation. Unlike other circulating biomarkers, CTCs provide information on DNA, RNA, and protein level alike [5,6,7]. Comprehensive downstream molecular and functional analyses can be carried out by isolated CTCs, which enables identification of both existing and new tumor biomarkers. Besides, isolating CTCs in a viable manner allows expansion of these cells in ex vivo cultures or xenograft models for further characterization of CTCs [8,9,10]. Therefore, the ability to detect and characterize these cells routinely could improve our current understanding of the underlying molecular changes in cancer during metastatic development and have profound effect on the management of the disease. However, the isolation of CTCs is challenging due to their extreme rarity in blood stream. While the peripheral blood cells (PBCs) are in the order of 10^9^, the number of CTCs is as low as one in one milliliter of blood [11]. Yet another challenge in the isolation of CTCs is their broad heterogeneity in their biological and physical properties, both within the patient and between patients [12,13,14,15].

Currently, a variety of techniques is available for CTC isolation from blood, each having its own advantages and inadequacies. These technologies can be basically classified in two groups: (i) those that are based on the differences in physical properties of CTCs and PBCs, including size, deformability, and electrical properties, and (ii) the others that are based on the differences in surface biological signatures [16,17]. Both methodologies can be either applied in a negative selection (eliminating the cells other than CTCs) or positive selection (selectively capturing/isolating the CTCs) manner [18]. Technologies that are based on the differences in physical properties of CTCs and PBCs apply either a mechanical filtration through porous filters or hydrodynamic enrichment that employs state-of the art microfluidic technologies [19]. These technologies offer the advantages of label-free isolation of CTCs from whole blood. On the other hand, technologies that utilize differences in surface biological markers use antibodies or aptamers for bioaffinity-based capture of target cells either immunomagnetically or by using antibody-coated microfluidic systems, in a negative or positive selection manner [20]. 

Microfluidic technologies improve the separation efficiency of the CTCs due to their innate properties, like small size that is comparable to the size of biological particles. Comparable sizes of the microfluidic features enable the separation of the CTCs solely based on hydrodynamic forces. Such examples include pinched flow fractionation [21], deterministic lateral displacement [22,23], or curved microchannels [24,25,26] that have been successfully adopted so far to isolate CTCs from blood. 

In this study, we present a novel technique to enhance the separation particularly by using spiral microfluidic channels for CTCs enrichment from blood. This has been achieved by integrating a hydrofoil structure at the downstream of the spiral channel (Patent pending, PCT/TR2019/050295). The hydrofoil increases the distance between the streams of CTCs and PBCs (white blood cells, WBCs in this case), which are already distributed about two focal axes by the spiral channel, thereby improving the performance. The performance of the system has been verified numerically and optimized experimentally. Analytical validation of the system has been carried out using CTC spiked blood samples from healthy donors. WBC depletion ratio, CTC recovery rate, and cell viability have been investigated. 

### 1.1. Principle of Particle Focusing in Spiral Microfluidic Channels

The hydrodynamic forces acting on a particle, arising from the parabolic velocity profile in the channel, interaction of the particle with the channel boundaries, and secondary flow induced by the curvature of the channel, focuses the particle at definite positions along a spiral microfluidic channel. Although underlying physics has been discussed in detail in [27], here we present the basics for completeness.

The net lift force (Equation (1)), which arises from the competition between shear gradient lift due to parabolic flow profile and wall lift force, cause particles to focus at certain equilibrium positions across a microchannel [28].

(1) $$F_{L}=\;{\frac{2\rho U_{avr}}{D_{H}{}^{2}}}^{2}a^{4}c_{L}$$

where *ρ* is the density of the carrier fluid, *U_avg_* is the average flow velocity in the channel, *a* is the particle diameter, *c_L_* is the lift coefficient, and *D_H_* is the hydraulic diameter of the microchannel [29]. However, several equilibrium positions exist for a given channel cross-section: in a rectangular channel, two equilibrium positions form near the top and bottom walls. The equilibrium position can be unified by a biasing Dean force [29] (Equation (2)), arising from Dean vortices formed in a spiral microchannel:

(2) $$F_{D}=3\pi \mu U_{D}a$$

where *µ* is the dynamic viscosity of the carrier fluid, and *U_D_* is the average velocity in the Dean vortex. In a Dean vortex, focusing positions substantially differ for different sized particles since the ratio of the lift force and the Dean force vary with the cube of the particle size (*F_L_*/*F_D_* ∝ *a^3^*). 

In a spiral channel, uninterrupted Dean force due to continuous curvature of the channel cause the particles to focus along the flow. However, the particles need to travel along a finite length of the spiral while migrating to the equilibrium position in the vortex, which limits the minimum length of the spiral [29]. 

### 1.2. Device Design and Simulations

**Figure 1:**
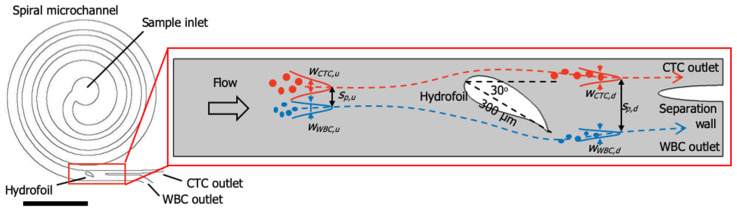
The hydrofoil located at the downstream of a spiral microchannel and the working principles of the hydrofoil. Circulating tumor cells (CTCs) (red dots) and white blood cells (WBCs) (blue dots) are pre-focused about their respective equilibrium positions separated at a distance of *s_p,u_* via a spiral channel at the upstream. The hydrofoil affects the stream, such as to increase the separation distance between the CTC and the WBC streams at the downstream to *s_p,d_*, which is large enough to direct the separated streams to CTC and WBC channels at the downstream separated by a separation wall. The length of the scale bar is 3 mm.

Here, we propose to use a hydrofoil located at the downstream of a spiral microchannel to facilitate the separation of the particles, particularly the circulating tumor cells (CTCs) from the white blood cells (WBCs) (Figure 1). The spiral microchannel is a 4-loop Archimedes’ spiral with 300 µm width and 100 µm depth and has 150 µm spacing between the loops. The design utilizes a cambered hydrofoil (NACA9730 airfoil profile) with chord line length of 300 µm, located in the stream at a non-zero attack angle *α* (*α* = 30° as shown in Figure 1 (Patent pending (PCT/TR2019/050295)). 

The spiral channel only causes the particles to get distributed about their respective equilibrium positions with a separation distance *s_p,u_*, and the hydrofoil enhances the separation distance between the CTC stream and the WBC stream to *s_p,d_* (Figure 1), which is adequate for collecting particles at the outlet from different channels by introducing a separation wall (subscripts *u* and *d* denote upstream and downstream, respectively). Therefore, an adequate separation distance, which can otherwise be obtained with a spiral channel having larger number of turns, can be obtained by using a relatively short spiral channel. Additionally, the hydrofoil pushes the CTC stream towards the inner wall of the spiral channel. Consequently, the width of the CTC outlet channel can be minimized, yielding smaller volume at the CTC outlet, and therefore, a more concentrated CTC suspension. 

As the performance metric, we adopted resolution, *R*, commonly used in chromatography applications [30]:

(3) $$R=1.18\frac{S_{p}}{w_{CTC}+w_{WBC}}$$

where *s_p_* is the distance between the location of the distribution peaks for CTC and WBC stream, and *w_CTC_* and *w_WBC_* are the widths of the CTC and WBC distributions, respectively, at half the height of the distribution peaks (Figure 1). *R* > 1 indicates that the distributions do not overlap, and a greater *R* value indicates an improved separation. The hydrofoil enhances the separation such that the resolution at the downstream of the hydrofoil (*R_d_*) is greater than the resolution at the upstream (*R_u_*).

**Figure 2:**
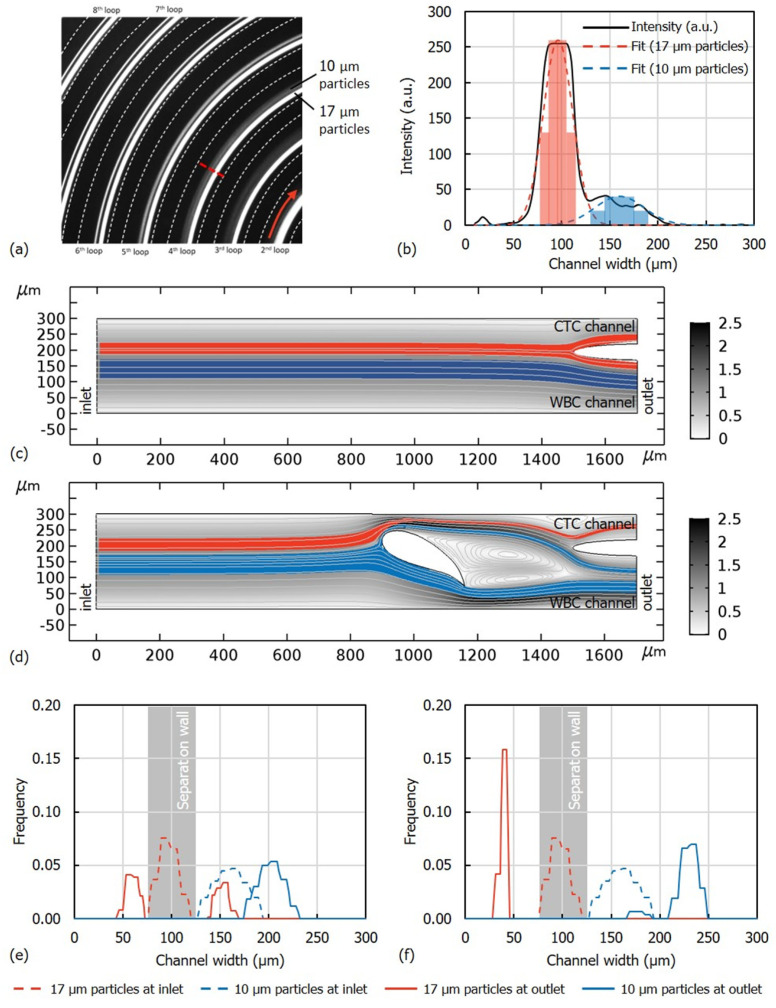
(**a**) Focusing of 10 µm and 17 µm fluorescent beads in a spiral channel with 8 loops. (**b**) Grayscale intensity profile of the beads across the channel at the 4th loop. Solid line denotes the measured intensity. Dash lines denote the Gaussian curves fitted to the intensity profile, to represent the individual distributions of the beads. Pale red and pale blue shaded regions denote the discretized distribution of the particles, as the input to the simulation model. Velocity field and the trajectories of the particles in the channel at the downstream of the spiral (Figure 1) (**c**) in the absence of the hydrofoil and (**d**) in the presence of the hydrofoil. Red and blue streams represent 17 µm and 10 µm particles, respectively. The scale bar indicates the flow velocity in m/s. Distribution of 10 µm and 17 µm particles at the inlet and the outlet (**e**) in the absence of the hydrofoil and (**f**) in the presence of the hydrofoil. The resolution at the upstream *R_u_* = 2.24 was increased to the resolution at the downstream *R_d_* = 30.46, which shows the effect of using hydrofoil to enhance separation.

To investigate the working principles, the part of the microfluidic channel shown in Figure 1 was modeled and simulated by using COMSOL Multiphysics^®^ 5.2a. In the model, distributions of 10 µm and 17 µm diameter particles representing WBCs and CTCs respectively were released from the upstream. To determine the distribution of particles at the upstream of the hydrofoil and their respective locations across the channel, flow of fluorescent beads with nominal diameter of 10 µm and 17 µm at a rate of 1000 µL/min was observed in an 8-loop spiral channel with the same cross-section. Figure 2a shows that the particles focusing become more prominent as they flow in the spiral channel. However, an apparent focusing of both 10 µm and 17 µm diameter could only be observed in the 7th loop, while in the former loops, the particles could only be distributed about their respective equilibrium positions. On the other hand, a hydrofoil, as illustrated in Figure 1, located at the downstream of a former loop would enhance the separation while reducing the total length of the channel, hence its hydrodynamic resistance. The distribution of the particles at the 4th loop was determined by analyzing the grayscale intensity across the channel (indicated by the red dash line in Figure 2a). Two peaks of the grayscale intensity profile across the channels at about 100 µm and 160 µm indicates the equilibrium positions of 17 µm and 10 µm diameter particles, respectively (Figure 2b). Two Gaussian curves were fitted to determine the distribution of the particles about these two equilibrium positions. These Gaussian curves were then discretized (pale blue and pale red shaded regions in Figure 2b) to resemble the distribution of the particles. Discretized distribution of 17 µm and 10 µm diameter particles representing the CTCs and WBCs respectively were then released from the inlet of the solution domain (Figure 2c,d), with resolution *R_u_* = 2.24 as calculated by referring to Equation (3). Firstly, the model without the hydrofoil was simulated as a reference (Figure 2c). A half-ellipsoid separation wall with 50 µm secondary diameter and whose center is placed 100 µm away from the upper (outer) wall of the channel has been employed as the separation wall. The separation wall is positioned along the length of the channel is just after the end tail of the vortex after the hydrofoil, to minimize the interference of the separation wall to the upstream fluid flow. The position along the channel width, on the other hand, has been selected both to minimize the volume at the CTC channel outlet (ratio of 75:175) and to maximize the CTC recovery rate. The results show that stream of 17 μm diameter particles was dissected at the separation wall and 55% of the particles were recovered from the WBC channel (Figure 2e). However, in the presence of hydrofoil, 100% of the 17 μm diameter particles could be recovered from the CTC channel (Figure 2f). Moreover, the resolution calculated at the downstream (outlet in Figure 2d) of the hydrofoil (*R_d_*) by referring to Equation (3) was 30.46 in comparison to the resolution at the upstream (*R_u_*) of 2.24, which proves the feasibility of utilizing a hydrofoil in separating the particles. It should be noted that the collection of all 17 µm diameter particles could also be achieved without the hydrofoil, only by panning the separation wall to the outer channel side by 25 µm; however, this increases the volume at the CTC channel outlet, decreasing the concentration of CTCs in suspension. 

## 2. Materials and Methods 

### 2.1. Device Fabrication

**Figure 3:**
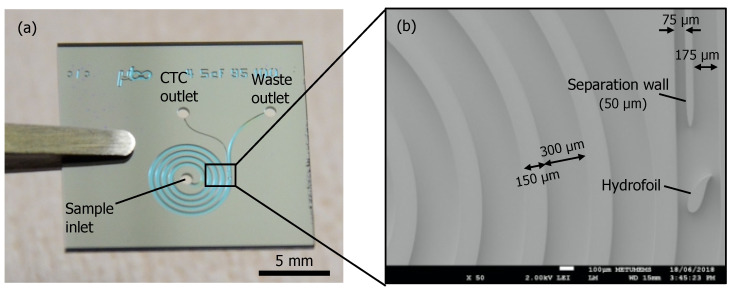
(**a**) Fabricated silicon/glass chip with four loops of a spiral channel and a hydrofoil at the downstream of the spiral channel. Channels were patterned by using deep reactive-ion etching (DRIE) of silicon and sealed with glass by anodic bonding. The CTC and waste outlets and the sample inlet are indicated. (**b**) SEM image of the section with the hydrofoil.

The microfluidic chips were fabricated with a silicon-glass stack microelectromechanical systems (MEMS) process where the microchannels and inlet and outlet ports were formed at the silicon side, and the glass side served as a transparent channel base. At the beginning, the silicon wafers went through a pre-cleaning with piranha and buffered hydrofluoric acid (BHF) cleaning steps. Then, the microfluidic channel pattern was formed on the active side of the silicon wafer by photolithography and deep reactive-ion etching (DRIE) dry plasma silicon etching down to the aimed depth (100 μm). After the channel formation, the wafers were cleaned for removing polymer residues and native silicon oxide by successive oxygen plasma, piranha and nitric acid, and BHF etch steps. Then, wafers were coated with 1 µm-thick thermal silicon dioxide. The silicon dioxide layer on the active side of the wafer serves as the etch protection layer while the one at the backside of the wafer form the masking layer for the next DRIE step. Afterwards, backside silicon dioxide was patterned with photolithography and reactive-ion etching (RIE) etching. This was followed by DRIE silicon etching through the whole wafer down to the channel opening correspondence at the active side, forming the inlet and outlet ports of the microfluidic device. After polymer removal and protection oxide strip, the silicon wafers were coated with thermal silicon dioxide again (0.3 µm), to act as a bio compatible insulation. Finally, the silicon wafers were anodically bonded to glass wafers and the wafer stacks were diced into chips. Figure 3 shows the fabricated chip and the scanning electron microscope (SEM) micrograph of the outlet section of the chip with the hydrofoil.

### 2.2. Microfluidic Setup

**Figure 4:**
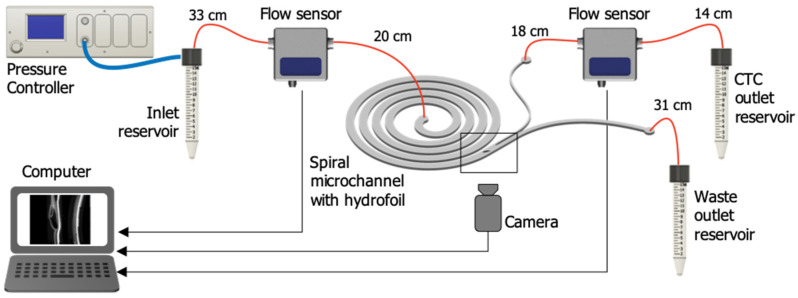
Test setup. A pressure-driven flow was generated inside the microfluidic chip using a pressure controller connected to pressurized N_2_. Two flow sensors were used for continuous measurement of the flow rate at the inlet and waste outlet of the chip. A custom-designed microfluidic chip holder houses the microfluidic chip and facilitates fluidic connections to the microchannel. An inverted fluorescent microscopy equipped with a highspeed camera connected to a computer were used for optical observation. Fused silica tubing was used to make fluidic connections (red lines).

A pressure-driven microfluidic setup was used to test the chips (Figure 4). The microfluidic setup consists of a pressure controller (Elveflow OB1 MK3, Paris, France) connected to pressurized N_2_ flow through a polyurethane tubing to generate the necessary pressure; two flow sensors (Elveflow MFS5, Paris, France) for continuous measurement of the flow rate at the inlet and waste outlet of the chip; a custom-designed microfluidic chip holder that houses the microfluidic chip and facilitates fluidic connections to the microchannel; fused silica tubing (inner diameter: 250 µm) (Postnova Z-FSS-250365, Landsberg am Lech, Germany); microfluidic fittings (Postnova Z-UC-F-123H, Z-UC-N-123-03X, Landsberg am Lech, Germany); polyurethane tubing (Elveflow Pneumatic connection kit, Paris, France); and inlet/outlet fluid reservoirs with reservoir adaptors (Elveflow, Paris, France).

### 2.3. Chip Conditioning

Before sample processing, chip conditioning has been carried out to prevent formation of air bubbles and biofouling. First, 1 mL of 96% ethanol (EtOH) was applied to the channels to prevent formation of air bubbles during fluid flow. The bubble-free microfluidic channels were then washed with 1 mL deionized (DI) water and 1 mL phosphate buffer solution (PBS). Then, the channels were pre-treated with Poly(L-lysine)-[g]-ploy(ethylene glycol) (PLL-g-PEG, 1 mg/10 mL in DI [31]) (SuSoS AG, Dübendorf, Switzerland) and incubated for 30 min to prevent biofouling. Then, the system was washed with 1 mL DI, then 1 mL PBS (not applied for the processes with microbeads) as the last step of conditioning.

### 2.4. Flow Rate Selection

Microbeads were used to select the optimum operation flow rates of the fabricated devices. A suspension of fluorescent microbeads with 10 µm and 17 µm diameter (Polysciences Europe GmbH, Hirschberg an der Bergstrasse, Germany) has been prepared in DI water with 100,000 bead/mL concentration for each size, with a total volume of 3 mL. Bead cluster formation was minimized by adding 0.5% Tween® 20 to the suspension. 

**Figure 5:**
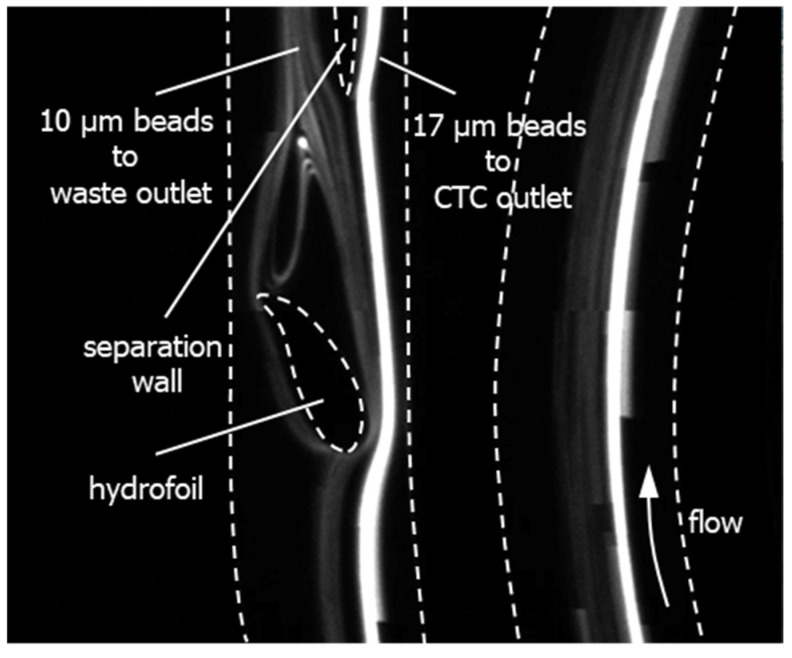
Visualization of the separation of beads at the hydrofoil region according to their sizes. Dash lines represent the boundaries of the flow.

The microbead mixture was separated into three equal-volume samples of 1 mL. After chip conditioning to prevent biofouling, samples were applied through the microchannel at volumetric flow rates of 1.0, 1.2, and 1.4 mL/min. The hydrofoil region of the microchannel was observed during these experiments and fluorescent streams corresponding to the paths followed by the beads were visualized (Figure 5). The samples were collected at the CTC outlet and waste outlet reservoirs. As the dead volume of the microfluidic setup (220 µl) is significant compared to the total volume of the sample (1 mL), recovery and purity rates were calculated from the number of beads collected at outlets, instead of incorporating inlet bead concentrations (Equations (4) and (5)). Three samples were taken from each reservoir for each experiment and the number of microbeads with 10 and 17 µm diameters were counted through the images generated by the automated cell counter (TC20, Bio-Rad Laboratories Inc., Hercules, CA, USA).

Two performance parameters were calculated by using the number of 10 µm and 17 µm microbeads at each outlet: the recovery rate and the purity of 17 µm diameter microbeads at the CTC outlet. These were calculated as follows:

(4) $$Recovery\;rate\;\left(\%\right)=100\left(\frac{\#\;of\;17\;µm\;beads_{CTC\;outlet}}{\#\;of\;17\;µm\;beads_{CTC\;outlet}+\;\#\;of\;17\;µm\;beads_{waste\;outlet}}\right)$$

(5) $$\begin{array}{c} Purity\;\left(\%\right)\\ =100\left(\frac{\#\;of\;17\;µm\;beads_{CTC\;outlet}}{\#\;of\;17\;µm\;beads_{CTC\;outlet}+\;\#\;of\;10\;µm\;beads_{CTC\;outlet}}\right) \end{array}$$

### 2.5. Cell Culture, Blood Collection, and Sample Preparation

Cultured human breast adenocarcinoma cell line (MCF-7, Leibniz-Institut DSMZ, ACC 115, Braunschweig, Germany) was used in spiking experiments as a CTC model. MCF7 cells were cultured in growth medium containing Dulbecco’s Modified Eagle’s Medium—High Glucose (DMEM-HG, Gibco, Thermo Fisher Scientific, 10569010, Waltham, MA, USA), 10% Fetal Bovine Serum (FBS, Biological Industries, 04-007-1A, Crowell, CT, USA), 1% Minimum Essential Medium (MEM) Non-Essential Amino Acids (Gibco, Thermo Fisher Scientific, 11140035, MA, USA) and 1% Penicillin-Streptomycin (Sigma-Aldrich, P4333, Darmstadt, Germany) at 37 °C with 5% CO_2_. The cells were passaged every 72 h. When the cells reached 70–80% confluency, they were detached from culture flask with trypsin-EDTA (0.25%, Sigma-Aldrich, T4049, Darmstadt, Germany) and resuspended in phosphate buffer saline (PBS). Cell Tracker Red CMTPX Dye (5 µM, Invitrogen, Thermo Fisher Scientific, C34552, MA, USA) was added into cell suspension, incubated for 25 min at 37 °C in dark, washed with PBS at 300× *g* for 5 min and diluted to appropriate concentration.

After dilution, cells (50–3500 cells/0.5 mL PBS) were spiked into whole blood (5 mL) collected from healthy donors. Ethical approval for blood collection was taken from the Ethical Committee of Middle East Technical University, Ankara Turkey, and studies performed were in accordance with the ethical regulations under ethical committee-approved protocols (Protocol No: 2018-FEN-043). Informed consent form, that is approved by the ethical committee, was obtained from each donor before participating to the study. Following spiking, double red blood cell (RBC) lysis was carried out to eliminate as many RBCs as possible. RBC Lysis Buffer (BioLegend, 420301, San Diego, CA, USA) was diluted to working concentration with deionized water and warmed to room temperature. RBC lysis buffer (100 mL) was added to whole blood-MCF-7 mixture and vortexed gently. The solution was incubated for 15 min at room temperature in dark. After incubation, cells were centrifuged at 350× *g* for 5 min and supernatant was discarded. Cell pellet was resuspended with RBC lysis buffer (10 mL), incubated for 5 min at room temperature in dark and centrifuged at 350× *g* for 5 min. The supernatant was discarded and the cell pellet was diluted with PBS (10 mL) and passed through a 30 µm cell strainer (Miltenyi Biotec, 130-098-458, Bergisch Gladbach, Germany). Cell concentration of the sample was counted with an Automated Cell Counter (BioRAD TC20, Hercules, CA, USA). This suspension containing white blood cells (WBCs) and spiked MCF7 cells was used for the analytical performance characterization of the microfluidic chip.

### 2.6. Analytical Performance Characterization

**Figure 6:**
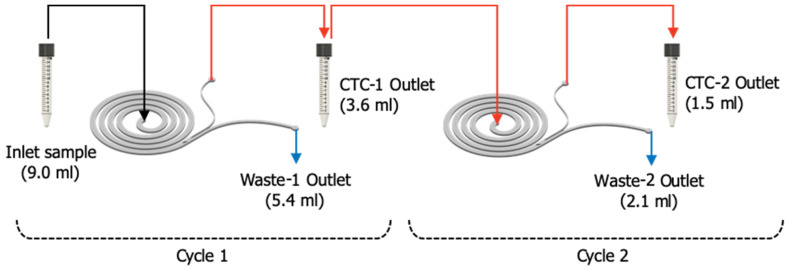
A schematic of the two-cycle sample processing through the same microfluidic chip.

Analytical performance characterization studies were carried out to set the CTC Recovery and WBC Depletion rates of the microfluidic chip. The studies were conducted using MCF7–WBC cell suspensions, prepared as explained in Section 2.5. 1 mL of the 10 mL cell suspension was separated to estimate the real number of spiked MCF7 cells, via optical counting, as detailed in Section 2.7. The remainder of the cell suspension (9 mL) containing white blood cells (WBCs) and MCF7 cells in various spiking ratios was applied to the microchannels with a volumetric flow rate of 1.2 mL/min—corresponding to ~1.7 bar applied pressure from the inlet and collected in CTC outlet containing enriched CTCs, and waste outlet. Figure 6 shows the schematic of the sample processing procedure used during the analytical performance characterization studies. Analytical data was collected for 1-Cycle and 2-Cycle processes and their performance was compared. In 2-Cycle processing, the sample collected at the CTC-1 outlet was further processed immediately after collection, with the same operational conditions and by using the same microfluidic chip. 

A total of 9.0 mL of the sample was passed through the system in the first cycle. As the output volume, 3.6 mL enriched sample was collected from CTC-1 outlet and 5.4 mL of sample was collected from waste-1 outlet. In the second cycle, 3.6 mL of enriched sample was passed through the system. The output volumes of two times enriched sample are 1.5 mL and 2.1 mL collected from CTC-2 outlet and waste-2 outlet, respectively. The volume ratio is about 2:3, which is directly proportional to the hydraulic resistance of the outlet channels. A total of 15 experiments were performed at spiking ratios between 50–3500 CTCs/mL with serial dilution, to cover a wide range of CTC numbers that might be found in patient blood. Lower spiking ratios were not covered because of higher error rates in spiking associated with serial dilution.

### 2.7. Analysis of WBC Depletion and CTC Recovery Rates

WBC concentrations in the inlet (through unprocessed 1 mL part of the sample) and outlet samples were calculated via automated cell counter. Since the numbers of WBCs are extremely higher than the number of spiked MCF7 cells (~4–6 orders of magnitude), it is assumed that the cell count gives the WBC concentration of the sample. The total number of cells in each suspension was estimated by multiplying the respective cell concentration with the volume of the suspensions. The rate of WBC depleted after each process was calculated as follows:

(6) $$Depletion_{Cycle\;1}\left(\%\right)=100\left(1-\frac{\#\;of\;WBC_{CTC\;outlet\;of\;Cycle\;1}}{\#\;of\;WBC_{inlet}}\right)$$

(7) $$\;Depletion_{Cycle\;2}\left(\%\right)=100\left(1-\frac{\#\;of\;WBC_{CTC\;outlet\;of\;Cycle\;2}}{\#\;of\;WBC_{inlet}}\right)$$

The rate of CTC recovery was calculated through optical inspection of the fluorescently stained MCF7 cells in the inlet (through unprocessed 1 mL part of the sample) and outlet cell suspensions. The cells collected from the outlets are centrifuged and resuspended in 1 mL PBS. Afterward, the cells are seeded into 96-well plates (100 µL/well) and centrifuged. The images of each well (acquired by stitching of 24 images/well) were taken under inverted fluorescent microscope (DMi8 Microscope, Leica Microsystems, Germany) equipped with motorized 3-plate stage. The image analysis to count the number of CTCs were carried out with a custom-developed software (Aurvis Ltd. Şti., Ankara, Turkey). Briefly, the python-based image processing software assembles 24 images of a well, adjusts the assembled image to concretize the MCF7 cells, and counts the stained cells. The counting process detects the red colored circular shapes and the GUI of the software enables the user to make changes after counting steps. After image analysis, CTC recovery rates were calculated as follows:

(8) $$Recovery\;rate_{Cycle\;1}\left(\%\right)=100\left(1-\frac{\#\;of\;MCF7_{waste\;outlet\;of\;Cycle\;1}}{\#\;of\;MCF7_{inlet}}\right)$$

(9) $$Recovery\;rate_{Cycle\;2}\left(\%\right)=100\left(1-\frac{\#\;of\;MCF7_{waste\;outlet\;of\;Cycle\;2}}{\#\;of\;MCF7_{inlet}}\right)$$

### 2.8. Viability Analysis

To investigate the effect of hydrodynamic forces generated during the passage of the cell suspension through the chip on the viability of CTCs, a pure MCF7 cell suspension was processed by the microfluidic chip using the same protocol explained above. Briefly, 3 mL of MCF7 cell suspension (10^6^ cells/mL) was passed through the chip. Total cell count and viability were measured at the inlet (before process) and outlets of the chip, after Cycle 1 and Cycle 2, and compared to the control group that are not processed but incubated in PBS throughout the experiment. Cell count and viability analysis were carried out by an automated cell counter using Trypan blue exclusion assay, by mixing 10 µL cell suspension with 10 µL Trypan blue (0.4%, Sigma-Aldrich, T8152, Darmstadt, Germany). Cell concentrations obtained from automated cell counter for each suspension were multiplied by the volume of inlet and outlet cell suspensions to calculate total and viable cell numbers. Experiments were carried out in triplicates. Total and viable cell loss was calculated as follows:

(10) $$Viable\;cell\;loss\;\left(\%\right)=100\left(\frac{\#\;of\;viable\;MCF7_{inlet}-\;\#\;of\;viable\;MCF7_{both\;outlets}}{\#\;of\;viable\;MCF7_{inlet}}\right)$$

(11) $$Total\;cell\;loss\;\left(\%\right)=100\left(\frac{\#\;of\;total\;MCF7_{inlet}-\;\#\;of\;total\;MCF7_{both\;outlets}}{\#\;of\;total\;MCF7_{inlet}}\right)$$

## 3. Results and Discussion

### 3.1. Flow Rate Optimization

Table 1 presents the average and standard deviation values of the recovery rate and purity of 17 µm diameter beads at the CTC outlet for different flow rates.

The results show that within the swept range the effect of flow rate on the recovery rate of 17 µm diameter microbeads is insignificant indicating that recovery rate can be maintained within a large operation flow rate range. However, the contamination of smaller sized particles is significantly higher for 1.0 mL/min flow rate. The purity is maximized at 1.2 mL/min flow rate as well as the recovery rate. To verify, flow rates were swept between ~0.75–1.5 mL/min also by using fluorescently stained MCF7 cells mixed with WBC. Fluorescent traces of MCF7 cells revealed that 1.2 mL/min is the optimum flow rate for the collection of MCF7 cells in CTC outlet. Therefore, this value has been selected as the operation flow rate for the rest of the studies. 

### 3.2. WBC Depletion and CTC Recovery

**Figure 7:**
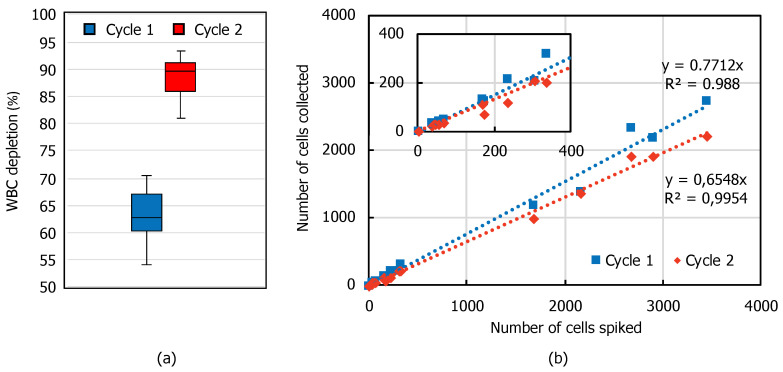
(**a**) A box-plot of WBC depletion (n = 15) and (**b**) linear fit of CTC recovery after first and second cycle of the spiral microfluidic chip process at different spiking rates (50–3500 CTCs/5 mL blood. Inset shows the zoomed view for spiking values < 400 CTCs.

Depletion of WBCs after one- and two-cycle spiral microfluidic chip process is shown in Figure 7a. After the first process, 63.1 ± 2.7% of WBCs that are initially present in the sample were removed, while after the second process, 88.3 ± 1.7% of the WBCs are eliminated, corresponding to ~10X enrichment of CTCs. Data represent the average of 15 experiments spanning MCF7 spiking between 50 and 3500. CTC recovery rates, as estimated from the linear fit of recovery rates obtained at each spiking experiment (Figure 7b), were calculated as 77.1 ± 4.8% for the first cycle and 65.5 ± 3.1% for the second cycle. The repeatability of the data is high (R^2^~0.99), showing that the recovery rate is independent of the spiked cell number. As expected, there is a trade-off between WBC depletion and CTC recovery. Application of Cycle 2 decreases the recovery rate as some of the MCF7 cells escape to the waste outlet in the second run. However, the WBC depletion increases to 88.3% in Cycle 2, improving the purity. Hence, for applications requiring higher purity, the sample can be processed in two cycles, while if the CTC recovery is more important, the process can be run once. 

The system is very high-throughput (1.2 mL/min volumetric flow rate), taking less than 9 min to complete Cycle 1 for 10 mL of inlet at the sample (corresponding to 5 mL of whole blood), and another 3 min for Cycle 2. The total sample preparation time and chip conditioning time is ~1 h, and combined with the sample processing time and cleaning processes, total duration of the process takes less than 2 h.

Spiral microfluidic chips for the enrichment of CTCs have been previously reported by several groups. Direct comparison of these studies with the current work is not possible due to specific design architectures; however, one advantage of the presented work over the reported ones is the high throughput operation with similar recovery rates, which is significant for the high-volume blood processing requirements of rare CTC isolation. Besides, the presented device simplifies the operation of spiral chip hydrodynamic focusing by eliminating the use of a sheath flow utilized in [24,32,33,34], to focus cells towards the outer channel. Moreover, the collected volume at CTCs outlet increases when sheath fluid is used, which necessitates additional concentration of collected cells for downstream analysis. Sheath-less high throughput spiral channels with trapezoidal cross-section were presented in [35,36]; however, the non-rectangular shape of these channels introduces fabrication challenges, especially for production scaling. 

### 3.3. Effect on Cell Viability

Although hydrodynamic CTC enrichment has the advantages of being label-free and requiring no external forces for operation, it has the major drawback of decreased cell viability due to high shear stress exerted on the cells. The hydrodynamic shear stress, which is the combination of fluid shear stress and extensional shear stress, creates a frictional force on the cells, resulting in decreased cell viability or even cell burst. As mammalian cells have no cell wall, they are prone to shear stress [37]. The detrimental effect of shear stress is related to several biological changes, including the induction of disarrangement of the cytoskeleton leading to cellular deformity, and the disintegration of the plasma membrane leading to cytoplasmic spillage and necrosis [38]. The type of the cell and duration and magnitude of the stress are important factors determining the level of destruction [39,40,41]. 

We have investigated the effect of hydrodynamic shear stress on MCF7 cells by analyzing total cell loss, viable cell loss, and percent viability at the outlets of the chip. Total cell loss and viable cell loss after Cycle 1 and Cycle 2 are shown in Table 2. The viability of the inlet MCF7 cell suspension was 81.6 ± 5.0%. In calculating the viable cell loss, this value is normalized to 100% and the percent of viable cell loss in Cycle 1 and Cycle 2 was calculated based on this value. The number of viable MCF7 cells decreases by 15.7 ± 8.5% after the Cycle 1, and 32.8 ± 10.2% after Cycle 2. In the control group, where the MCF7 cells were incubated in PBS throughout the process, cell loss was negligible (3.3 ± 2.3%). Almost the same ratio was observed in total (viable + non-viable) cell loss after each process, indicating that the chip process has the same effect on the loss of viable or non-viable cells. A portion of these loses may be attributed to the downstream processes like centrifugation, recollection, etc. However, most of them result from the damage caused by fluid shear stress. We have applied a 1.2 mL/min volumetric flow rate corresponding to a shear stress of approximately 40 dyn/cm^2^ in the cross section of the microfluidic channel before the hydrofoil. The shear stress further increases in the channel after the hydrofoil and the separation wall as the channel dimensions decreases. The average shear stress range in the blood vessels is 0.5–30 dyn/cm^2^, at which most of the CTCs undergo apoptosis or necrosis, while the surviving cells develop resistance mechanisms to circumvent the shear-stress induced cellular damage [42,43]. A higher loss in Cycle 2 also indicates increased fragility of the cells after Cycle 1. The effect of hydrodynamic shear stress on mammalian cells viability has been investigated in several studies using similar shear stress-dominated microfluidic systems. Using a spiral microfluidic chip developed for isolation of canine cutaneous mast cell tumor (MCT), Ketpun et al. [38] have analyzed the effect of exposure to hydrodynamic shear stress on the viability of MCT. Results show that 40% of the viable cells are lost during the process due to necrosis. Although cell loss is observed to an extent, our results indicate that most of the cells remain viable even after two cycles of process. 

Results also indicated that viabilities of cells collected at the CTC outlet are significantly higher than those obtained at the waste outlet, for both processes (Table 3). Due to the effect of shear stress on plasma membrane integrity, cytoplasmic spillage and even loss of nucleus may take place [38]. Loss of cytoplasmic content results in decreased cell size and the collection of damaged cells in the waste outlet; while the healthy and viable MCF7 cells, which survive the shear stress and preserve their membrane integrity, also preserve their size and tend to flow to the CTC outlet. 

**Figure 8:**
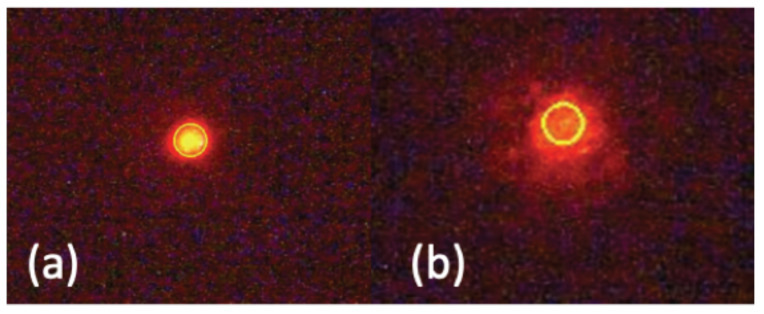
Fluorescent microscopy images of the cells collected at (**a**) CTC outlet and (**b**) waste outlet. CTCs collected at the waste outlet have lost the integrity, as exemplified by the irregular shape of the cell in (**b**). Yellow circles are overlaid by the utilized cell counting software.

This was confirmed by the optical inspection of the MCF7 cells collected in both outlets (Figure 8). Cells collected in the CTC outlet are mostly intact and circular, while the most of the cells collected in the waste outlet seem disintegrated.

## 4. Conclusions

Tremendous efforts were put forward for the isolation of CTCs within the last two decades, especially for the development of microfluidic-based methods. However, no single method can satisfy all the expectancies, like high sensitivity and specificity, viable cell isolation, ease of use, and cost efficiency, that present the impediments for the use of CTC technologies routinely in clinical settings. This is mainly due the selection biases applied either on the size of the cells or their immunological differences, not to mention the rarity of CTCs being the major challenge in high precision CTC isolation. In this study, we present a novel method to improve the separation efficiency of CTCs by using inertial microfluidics, which provides label-free isolation of cells. By integrating a hydrofoil structure at the downstream of a spiral channel, the resolution between the streams of CTCs and WBCs has been increased and the collected sample volume is decreased. 

We have demonstrated the method for the enrichment of MCF7 cells from spiked blood samples. However, the method can be adapted to design microfluidic chips for the enrichment of specific CTCs with predictable size. For this purpose, firstly a spiral channel should be designed for pre-focusing the cells, in a way that the resolution calculated by using Equation (3) is approximately 1. This can be achieved by referring to comprehensive experimental data for different sized particles available in the literature [44]. Then, an asymmetric hydrofoil should be located at the downstream of the spiral channel such that the resolution at the downstream of the hydrofoil is around 10 or larger. Lastly, collection channels should be located such as to recover the CTCs at the downstream of the hydrofoil.

Analytical validation studies have been carried out by both single cycle and two cycle processing of the MCF7-spiked blood sample. Application of Cycle 2 improves the purity by increasing the WBC depletion with the expense of decreased CTC recovery. Viability ratio of the collected MCF7 cells were not affected by the enrichment process. The enrichment process is very high-throughput (1.2 mL/min volumetric flow rate), taking less than 9 min to complete Cycle 1 for 10 mL of inlet at the sample and another 3 min for Cycle 2. For applications requiring higher purity, the sample can be processed in two cycles. Single step process can be applied if the CTC recovery is more important. Clinical studies are being carried out to validate the clinical performance of the system with different cancer types together with downstream molecular analysis to show the clinical utility. 

## Figures and Tables

**Table 1:** The recovery rate and purity of 17 µm diameter beads at the circulating tumor cells (CTC) outlet at different flow rates. Experiments were carried out in triplicates and average recovery rates and purities were given with standard deviations (STD).

Flow Rate (mL/min)	1.0	1.2	1.4
**Recovery rate ± STD (%)**	99.0 ± 1.7	99.1 ± 1.6	98.1 ± 0.2
**Purity ± STD (%)**	79.2 ± 5.8	97.7 ± 0.3	96.2 ± 1.7

**Table 2:** Effect of hydrodynamic forces on cell viability as shown by viable cell loss and total cell loss after first and second cycle of the spiral microfluidic chip. Data represent the average of 3 experiments ± Standard error of the mean (SEM).

	Viable Cell Loss ± SEM (%)	Total Cell Loss ± SEM (%)
**Cycle 1**	15.7 ± 8.5	15.3 ± 7.5
**Cycle 2**	32.8 ± 10.2	29.7 ± 9.3
**Control**	3.3 ± 2.3	4.7 ± 2.3

**Table 3:** Viability of cells collected from CTC and waste outlets after Cycle 1 and Cycle 2 of the process. Data represent the average of three experiments ± Standard error of the mean (SEM).

	Inlet	Cycle 1		Cycle 2	
		CTC outlet	Waste outlet	CTC outlet	Waste outlet
**Viability ± SEM (%)**	81.7 ± 2.9	84.7 ± 2.3	62.5 ± 6.9	85.2 ± 1.6	73.5 ± 3.8
